# E6 and E7 from Beta Hpv38 Cooperate with Ultraviolet Light in the Development of Actinic Keratosis-Like Lesions and Squamous Cell Carcinoma in Mice

**DOI:** 10.1371/journal.ppat.1002125

**Published:** 2011-07-14

**Authors:** Daniele Viarisio, Karin Mueller-Decker, Ulrich Kloz, Birgit Aengeneyndt, Annette Kopp-Schneider, Hermann-Josef Gröne, Tarik Gheit, Christa Flechtenmacher, Lutz Gissmann, Massimo Tommasino

**Affiliations:** 1 DKFZ, Heidelberg, Germany; 2 International Agency for Research on Cancer, World Health Organization, Lyon, France; 3 Department of Pathology, University Hospital of Heidelberg, Heidelberg, Germany; 4 Department of Botany and Microbiology (honorary member), King Saud University, Riyadh, Saudi Arabia; University of Wisconsin, United States of America

## Abstract

Cutaneous beta human papillomavirus (HPV) types appear to be involved in the development of non-melanoma skin cancer (NMSC); however, it is not entirely clear whether they play a direct role. We have previously shown that E6 and E7 oncoproteins from the beta HPV type 38 display transforming activities in several experimental models. To evaluate the possible contribution of HPV38 in a proliferative tissue compartment during carcinogenesis, we generated a new transgenic mouse model (Tg) where HPV38 E6 and E7 are expressed in the undifferentiated basal layer of epithelia under the control of the Keratin 14 (K14) promoter. Viral oncogene expression led to increased cellular proliferation in the epidermis of the Tg animals in comparison to the wild-type littermates. Although no spontaneous formation of tumours was observed during the lifespan of the K14 HPV38 E6/E7-Tg mice, they were highly susceptible to 7,12-dimethylbenz(a)anthracene (DMBA)/12-0-tetradecanoylphorbol-13-acetate (TPA) two-stage chemical carcinogenesis. In addition, when animals were exposed to ultraviolet light (UV) irradiation, we observed that accumulation of p21^WAF1^ and cell-cycle arrest were significantly alleviated in the skin of Tg mice as compared to wild-type controls. Most importantly, chronic UV irradiation of Tg mice induced the development of actinic keratosis-like lesions, which are considered in humans as precursors of squamous cell carcinomas (SCC), and subsequently of SCC in a significant proportion of the animals. In contrast, wild-type animals subjected to identical treatments did not develop any type of skin lesions. Thus, the oncoproteins E6 and E7 from beta HPV38 significantly contribute to SCC development in the skin rendering keratinocytes more susceptible to UV-induced carcinogenesis.

## Author Summary

Epidemiological and biological lines of evidence support a possible involvement of a sub-group of human papillomaviruses (HPV), referred to as cutaneous beta HPV types, in the development of non-melanoma skin cancer (NMSC). However, their role in carcinogenesis, in particular whether they synergize with other NMSC risk factors, e.g. UV irradiation, is still unclear. Here, we describe the generation of a novel model of transgenic mice (Tg) expressing the viral oncoproteins E6 and E7 from cutaneous beta HPV38 in the basal layer of the epidermis. We established two independent lines of HPV38 E6/E7 Tg mice and showed that they both have an increased susceptibility to develop squamous cell carcinoma (SCC) in comparison to the wild-type animals when exposed to chemical carcinogens and UV irradiation. Most interestingly, we found that UV irradiation of the Tg animals, promoted the formation of skin lesions that closely resembled the SCC-precursor lesions in humans, actinic keratosis and subsequently SCC. In contrast, we observed that wild-type mice developed neither actinic keratosis nor SCC when exposed to the same dose of UV. In conclusion, we present evidence that supports the role of cutaneous beta HPV types in skin carcinogenesis.

## Introduction

Non-melanoma skin cancer (NMSC) is the most common cancer in adult fair-skinned populations [1]. Ultraviolet light (UV) is a key risk factor for the NMSC [2]–[4]. In addition, it appears that infectious agent(s) may favor skin carcinogenesis. This is suggested by the fact that immuno-compromised organ transplant recipients (OTRs) have a 50–100-fold higher risk of developing NMSC compared to the general population [5], [6] [6] [7] [8]. A sub-group of cutaneous human papillomavirus (HPV) types, belonging to the genus beta of the HPV phylogenetic tree, are putative etiological factors of NMSC [9] [10]. These HPV types were first isolated in individuals with an autosomal recessive disorder, termed epidermodysplasia verruciformis (EV). EV individuals are susceptible to infection by beta HPV types and have a propensity to develop confluent flat warts, which, in approximately 30% of the cases, progress to squamous cell carcinomas (SCC) on sun-exposed areas [9] [10]. Accordingly, DNA from several beta HPV types was found in a high percentage of precursor lesions, actinic keratoses, and SCC from OTRs [11] [12] [13]. More recent studies indicate that beta HPV types are also involved in skin carcinogenesis in the normal population. Detection of antibodies against the major capsid protein L1 showed an increased seroreactivity to beta HPV types in patients with cutaneous SCC in comparison to healthy individuals [14] [15] [16] [17] [18] [19].

Functional studies have provided further evidence of an association of beta HPV types with NMSC. Since previous studies demonstrated the key role in cellular transformation of E6 and E7 oncoproteins from cervical cancer-associated mucosal high-risk (HR) HPV types, functional investigations on beta HPV types focused on the characterization of E6 and E7 biological properties. These studies showed that E6 and E7 from beta HPV types also displayed transforming capability in *in vitro* and *in vivo* experimental models [10]. E6 from beta HPV types associates with the pro-apoptotic protein Bak, a member of the Bcl-2 family, promoting its proteasomal degradation and preventing apoptosis in response to genomic stress [20] [21]. Studies from our group have shown that E6 and E7 from beta HPV38 are able to immortalize primary human keratinocytes [22] [23], similarly to E6 and E7 from the mucosal HR HPV types. Accordingly, we observed that HPV38 E6 and E7 expression in these cells leads to the accumulation of ΔNp73α, which in turn alters the p53 transcriptional functions [24].

Tg mouse lines expressing the entire early region of beta HPV8 (E6, E7, E1 E2 and E4 genes) or the E6 gene alone driven by the K14 promoter, spontaneously developed multifocal skin tumours and, in approximately 6% of the cases, SCC [25] [26]. In addition, a single dose of UV rapidly promoted papillomas and SCC formation [26]. In another study, Tg mouse models for the beta HPV20 and the benign cutaneous HPV27 were generated, in which E6 and E7 oncoproteins were expressed as single polycistronic transcript under the control of the K10 promoter that is active in the supra-basal differentiated layer of the skin epidermis [27]. Both Tg models developed skin lesions, including SCC after exposure to UV irradiation. However, no significant difference in the skin tumour incidence was observed between HPV 20 and 27 Tg animals [27]. Based on our *in vitro* data [22] [23] [24], we generated K10 HPV38 E6/E7 Tg mice [28]. These animals displayed hyperplastic and dysplastic patches in the skin epidermis, but no spontaneous development of skin cancer was observed during their life span. Application of the two-stage skin carcinogenesis protocol led to a strong increase in skin tumour incidence [28]. However, chronic UV irradiation of K10 HPV38 E6/E7 Tg did not lead to development of any type of skin lesions (Dong et al. unpublished data). The failure of HPV38 E6 and E7 to cooperate with UV irradiation in skin tumour development in this animal model could be explained by the fact that the viral genes were expressed in the suprabasal layers of the epidermis, while in humans beta HPV types infect and initiate the transcription of the early genes in the basal layer. To explore this hypothesis, we developed a novel transgenic mouse model for HPV38 with K14 promoter-driven expression of E6 and E7 in the basal and proliferative rather then the differentiated compartment of skin epidermis. Here, we show that ectopic HPV38 E6 and E7 expression in this location strongly enhances the susceptibility to chemical- and UV-induced carcinogenesis. Most importantly, chronic UV irradiation of K14 HPV38 E6/E7 Tg mice results in the development of actinic keratosis-like lesions and SCC, closely resembling the scenario observed in humans.

## Results

### Generation and characterization of K14 HPV38 E6/E7-Tg mice

**Figure 1:**
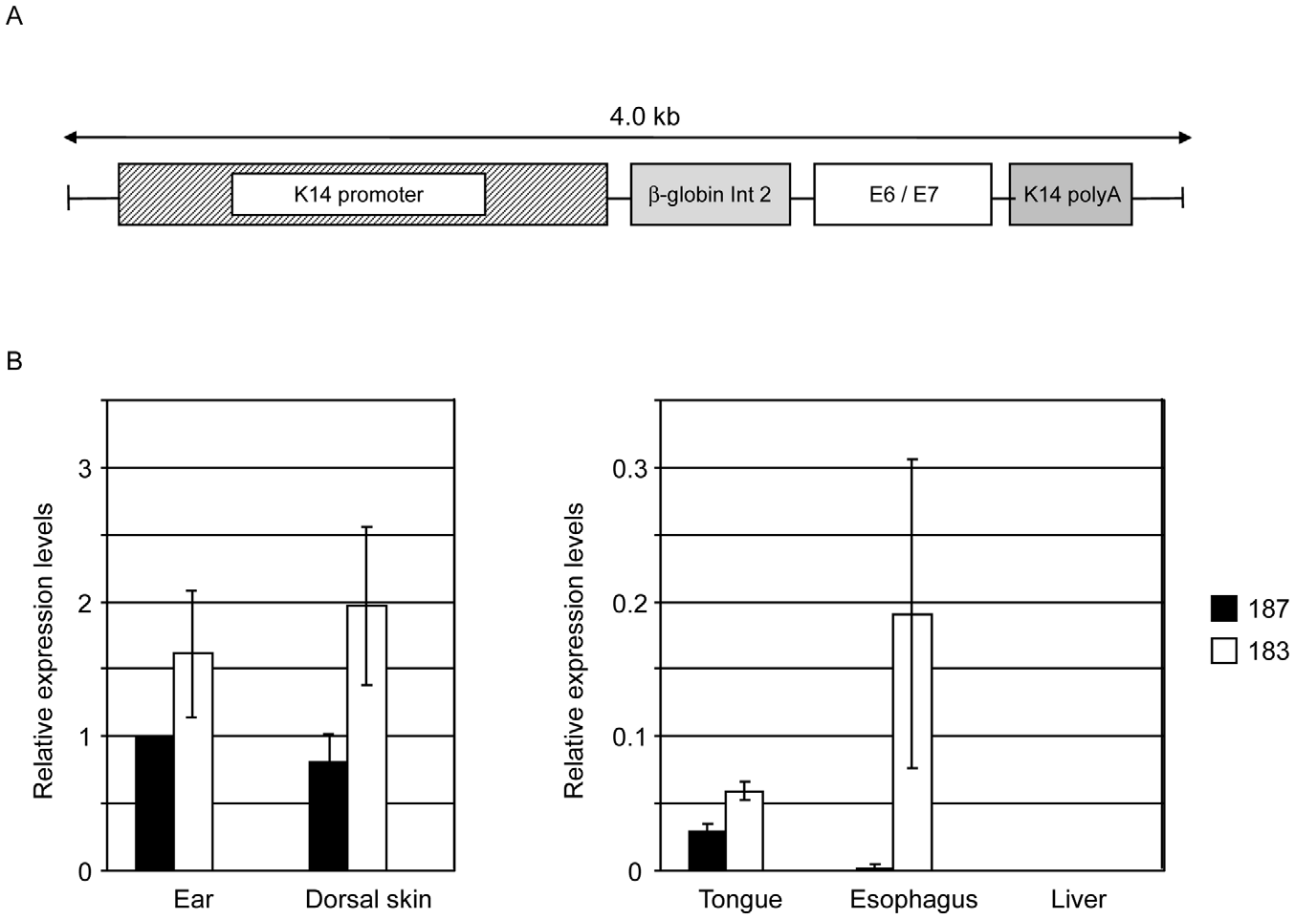
HPV38 E6 and E7 expression in Tg mice. (A) Schematic representation of the K14-HPV38 E6/E7 construct. (B) HPV38 E6 and E7 transcripts are differentially expressed in the epithelia of the two hemizygous Tg mouse lines 183, and 187. Total RNA was extracted from the ear, the skin, tongue, esophagus, and liver. After preparation of cDNA, E6 and E7 expression was determined by RT-qPCR and normalized towards the expression level of GAPDH. The data shown in the Figures are the means ±SD of three independent experiments. In each experiment the 187 ear data is set to 1 and the other values are consequently resized.

To evaluate the transforming properties of E6 and E7 from HPV38 in the proliferative compartment of skin epidermis, we generated Tg mouse lines expressing the two viral oncogenes under the control of the human keratin 14 promoter that is active in the basal layer of the epidermis [29]. A schematic representation of the transgene construct used is shown in Figure 1A. Transgene-positive offspring were identified by PCR of tail DNA using HPV38 specific primers. Two independent Tg mouse lines (183 and 187) were identified and bred successfully. Viral oncogene expression was determined by RT-qPCR in different epithelia, i.e. ear, dorsal skin, tongue, and esophagus. Line 183 expressed higher HPV38 E6/E7 levels than line 187 in all four examined epithelia (Figure 1B). In each Tg line, HPV38 E6/E7 expression also differed in the four epithelia, being highest in the dorsal skin and the ear, and comparably low in the tongue and esophagus (Figure 1B). As expected, no viral oncogene expression was observed in liver tissue that was included as a negative control (Figure 1B). No HPV38 E6 and E7 expression was detected in the same tissues of the wild-type animals (data not shown).

### HPV38 E6 and E7 induce cellular proliferation in the epidermis of Tg mice

**Figure 2:**
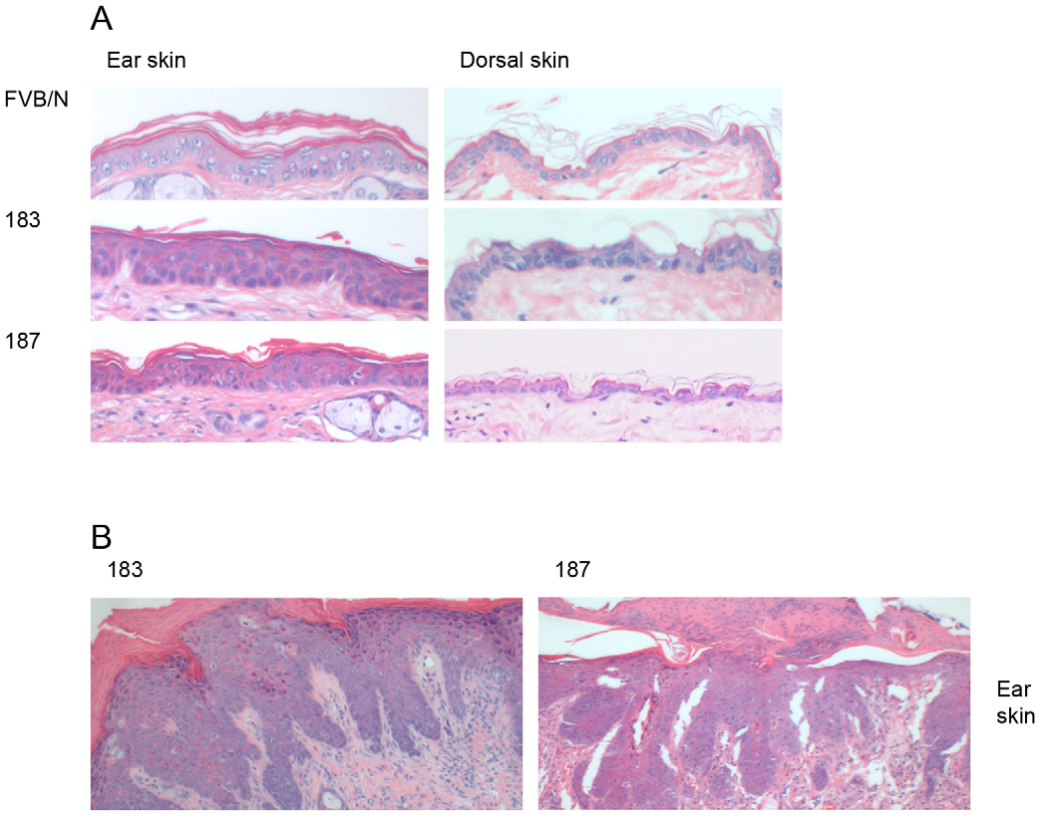
Histological analysis of skin specimens from wild-type FVB/N and Tg mouse lines. Representative pictures (original magnification 40×) of HE-stained sections of paraffin-embedded tissues are shown: (A) ear (left panel) and dorsal skin (right panel) of wild-type FVB/N and Tg mice of the lines 183, and 187. (B) Dysplastic ear skin of K14 HPV38 E6/E7-Tg mice.

Next, we examined whether HPV38 E6/E7 expression induced morphological alterations in the epithelia analyzing HE-stained sections of skin, ear, tongue and esophagus of FBN/V and K14 HPV38 E6/E7-Tg mice. Epidermal hyperplasia in the ear skin was observed in approximately 5% of 6–8 week-old mice from both Tg lines, as representatively shown in Figure 2A. These alterations, although also detected, were much less evident in dorsal skin (Figure 2A). No significant morphological changes were observed in epithelia of the esophagus and tongue of both Tg lines (data not shown).

The morphological alterations observed in ear skin were even more severe in older animals of lines 183 and 187. Approximately 10–15% of 12-month-old mice from both lines presented dysplasia and, hyperkeratosis. A representative section is shown in Figure 2B, where severe dysplastic keratinocytes, hyperkeratosis, endophytic papillomatous epidermis and a pronounced inflammation could be observed.

**Figure 3:**
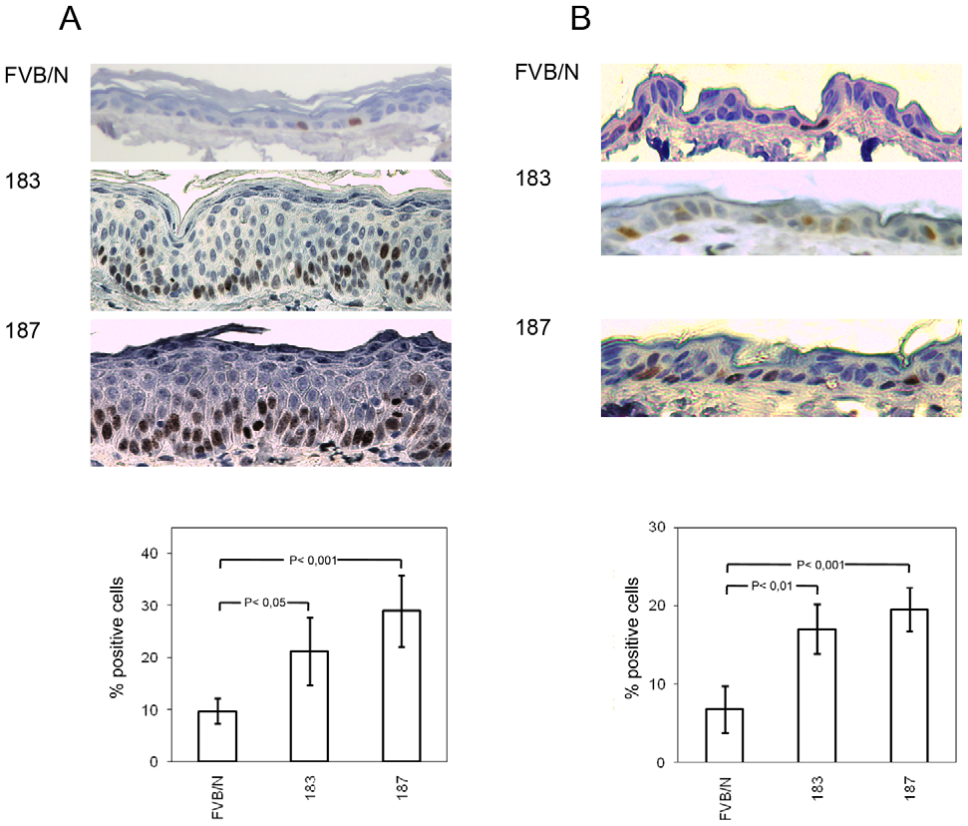
Analysis of cellular proliferation in the ear and dorsal skin of wild-type and transgenic mice. (A and B, top panels) Representative pictures of Ki-67 immunostained sections of paraffin-embedded ear skin and dorsal skin from wild-type (FVB/N) and Tg animals (lines 183 and 187). (A and B, lower panels) Quantification of Ki-67-positive cells (brownish signal) in wild-type and Tg epidermis was done by counting 400 hematoxylin-stained cells under 40× magnification in 4 different fields of epidermis. Differences between the Ki-67-positive cells in the HPV38 E6/E7 Tg mice (lines 183 and 187) versus the FVB/N mice were statistically significant as determined by Student's t-test with Welch correction for unequal standard deviation.

To determine whether the expression of HPV38 E6 and E7 oncoproteins resulted in a deregulation of cellular proliferation, we next analysed the expression of the proliferation marker Ki-67 by immunohistochemistry. A significant increase of Ki-67 positivity was observed in the ear (line 187 Vs FVB/N *p*<0,001, line 183 Vs FVB/N *p*<0.05) and dorsal skin (line 187 Vs FVB/N *p*<0.001, line 183 Vs FVB/N *p*<0.01) epidermis of the two Tg mouse lines (Figures 3A and 3B). Although morphological changes were not observed in tongue and esophagus up to the age of 12 months, an increased Ki-67 index was detected in the epithelia of these tissues at similar levels to those observed in the ear and dorsal skin (data not shown). To corroborate these data, we determined the levels of the positive cell cycle regulator cyclin A in protein extracts from dorsal skin of wild-type and Tg mice by immunoblotting. Cyclin A levels were higher in the two Tg mouse lines as compared to the control animals (data not shown).

Together, these data show that ectopic overexpression of HPV38 E6 and E7 oncogenes in the basal layer of the mouse epithelia significantly increased cellular proliferation.

### Enhanced formation of SCC in skin of K14 HPV38 E6/E7 transgenic mice upon DMBA/TPA treatment

**Figure 4:**
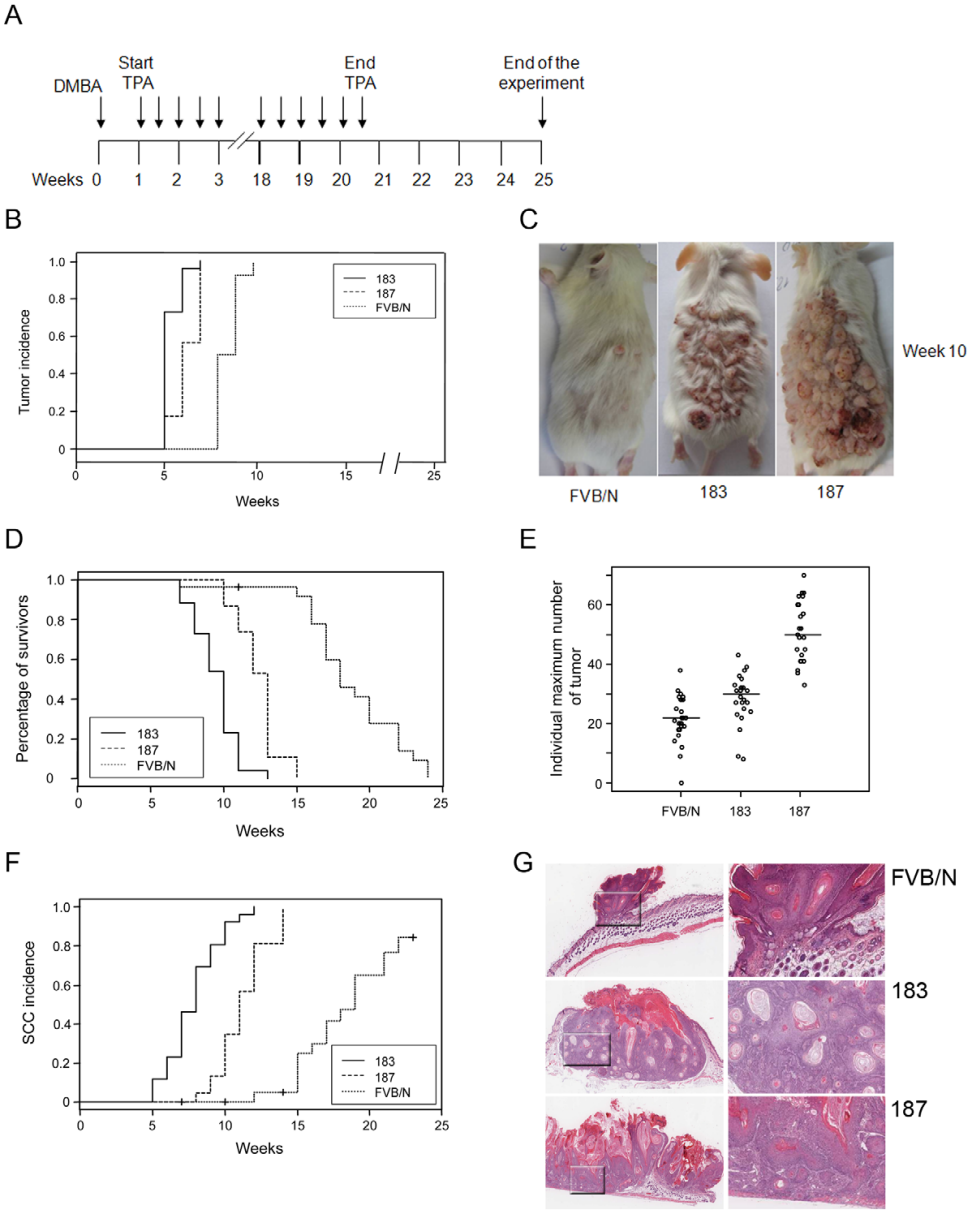
Tumour burden in wild-type and transgenic mice after DMBA/TPA treatment. (A) Schematic diagram of the initiation and promotion protocol using DMBA as initiator and TPA as promoter of the two-stage skin carcinogenesis approach. (B) Tumour incidence. Percentage of animals with skin tumours in the group of FVB/N wild-type and Tg cohorts of lines 183 and 187. Skin tumour formation was recorded each week until the end of the experiment in week 24 after the beginning of promotion. The difference between the curves of control and transgenic mice is statistically significant (*p*<0.0001 determined by logrank test for group data). (C) Representative pictures of dorsal skin from wild-type FVB/N and Tg mice 10 weeks after the beginning of tumour promotion. (D) Survival curves for DMBA/TPA-treated wild-type FVB/N and Tg animal cohorts of lines 183 and 187. Mice were sacrificed when putative SCC skin lesions reached the size of 15 mm in diameter. The difference between the curves of control and transgenic mice is statistically significant (*p*<0.0001 determined by logrank test for group data). (E) Tumour multiplicity. Maximum number of tumours per animal in the groups of wild-type and Tg lines. The number of tumours was recorded every week. Differences between the tumour multiplicity in the group of wild-type and Tg lines are statistically significant (control versus line 183, *p*<0.001; control versus line 187, *p*<0.001 as determined by Wilcoxon rank sum test). (F) Incidence of cutaneous SCC in the group of wild-type and transgenic mice. SCC in the sacrificed wild-type and Tg animals were confirmed by histological analyses. The difference between the curves of control and transgenic mice is statistically significant (*p*<0.0001 determined by logrank test for group data). (G) Representative pictures of HE-stained skin lesions from wild-type (FVB/N) and K14 HPV38 E6/E7-Tg mice (lines 183 and 187) collected after 10 weeks of chemical carcinogens treatment (original magnification 5×). Magnified areas are shown in the right panels.

Previous studies reported an increased incidence of papillomas and SCC in Tg mouse models expressing E6 and E7 from human or animal papillomaviruses [28] [30] [31] when exposed to chemical carcinogens. Therefore, we compared tumour susceptibility of wild-type and K14 HPV38 E6/E7 Tg mice in the multi-stage skin carcinogenesis protocol using DMBA as initiator and TPA as tumour promoter. Wild-type and Tg animals were exposed to a single treatment of DMBA followed by repeated TPA treatments for 20 weeks and subsequent examination for a further five weeks (Figure 4A). Seven weeks after initiation, 100% of Tg animals of both lines had developed tumours, while at the same time wild-type animals showed no skin lesions and became 100% tumour positive three weeks later (Figure 4B). At week 10, the DMBA/TPA-treated back skin of Tg mice was entirely covered by tumours, in contrast to the wild-type animals that developed only a small number of skin lesions (Figure 4C). Due to the high number of skin lesions including large and multiple SCC per mouse, all Tg animals of lines 183 and 187 were sacrificed at week 12 and 14, respectively, while this event was delayed until week 24 in the wild-type group (Figure 4D). The number of tumours per animal was significantly higher in the Tg cohort as compared to the wild-type cohort (Figures 4E). After completion of the experiment at week 24, histological examination of skin lesions from all control and Tg mice was performed. Accordingly, Tg mice of both lines developed SCC more rapidly (Figure 4F) and at higher incidence than the wild-type mice (Figure 4F). Representative sections of the tumours at week 10 from control and Tg animals are shown in Figure 4G. The shown tumour of the control animal was at an early stage and disruption of the basement membrane and extension of tumour islands into the underlying dermis started to be visible (Figure 4G). The representative sections from Tg animals evidenced SCC characterized by tumour cells with prominent intercellular bridges, abundant eosinophilic cytoplasm and a large and vesicular nucleus, plus aberrant accumulations of keratin (keratin pearls) (Figure 4G, line 183), as well as by irregularly elongated rete pegs with atypia, defined as vacuolization and nuclear abnormalities of cells of the stratified squamous epithelium invading the connective tissue (Figure 4G, line 187).

In summary, these results show that ectopic expression of HPV38 E6 and E7 in the proliferative compartment of skin epidermis significantly increases the tumour burden including papillomas and SCCs in a DMBA/TPA multi-step skin carcinogenesis approach.

### Reduced UVB-induced cell-arrest and enhanced UVB carcinogenicity in K14 HPV38 E6/E7 Tg mice

**Figure 5:**
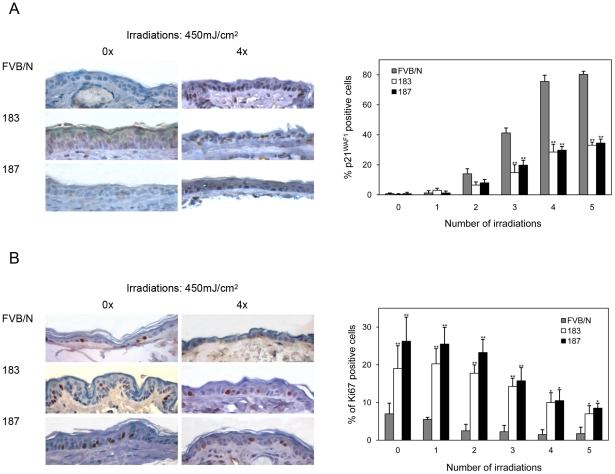
p21^WAF1^ and Ki-67 levels in the skin of wild-type and K14 HPV38 E6/E7-Tg mice after UVB irradiation. Wild-type and Tg animals were irradiated up to 5 times as described in Materials and Methods. 24 hours after the last irradiation, mice were sacrificed and skin tissue was analyzed by immuno-histochemistry. (A) Representative Ki-67 and p21^WAF1^ immunostainings of skin from wild-type and Tg mice non-exposed (0×) or four time (4×) exposed to UVB. (B) Quantification of p21^WAF1^ and Ki-67-positive cells in skin of wild-type and Tg mice before and after UVB irradiation. The percentage of p21^WAF1^ and Ki-67-positive cells in the epidermis was determined as described in the legend of Figure 3. The differences between the percentages of p21^WAF1^ or Ki-67-positive cells in the HPV38 E6/E7 Tg mice (lines 183 and 187) versus the FVB/N non-Tg mice are statistically significant (* = *p*<0.05, ** = *p*<0,001) as determined by Student's t-test.

UV irradiation is a key risk factor for NMSC in humans. Therefore, we next determined whether K14 HPV38 E6/E7-Tg mice had an enhanced susceptibility to UVB irradiation. Induction of DNA damage by UV irradiation normally leads to the activation of cellular defense mechanisms, mainly mediated by p53 activation that in turn induces cell cycle arrest prior to the S phase or apoptosis. The cell cycle block is primarily mediated by accumulation of the cyclin-dependent kinase inhibitor (CDK) p21^WAF1^, whose gene is positively regulated by p53. Short-term UVB irradiations of the skin of wild-type mice resulted in an accumulation of p21^WAF1^ in keratinocytes of the basal layer of the epidermis (Figure 5A). In contrast, this phenomenon was significantly less evident in the skin of Tg animals (Figure 5A, right panel, P<0,001 after the third UVB irradiation). In agreement with the p21^WAF1^ expression levels, staining of the Ki-67 proliferative marker was stronger in the skin of the Tg mice in comparison to the wild-type animals, even after several doses of UV irradiation (Figure 5B). These data show that HPV38 E6 and E7 have the ability to interfere with the regulation of cellular checkpoints activated by genomic stress, such as UV-induced DNA damage. Thus, it is likely that HPV38 enhances the carcinogenicity of UV irradiation.

**Figure 6:**
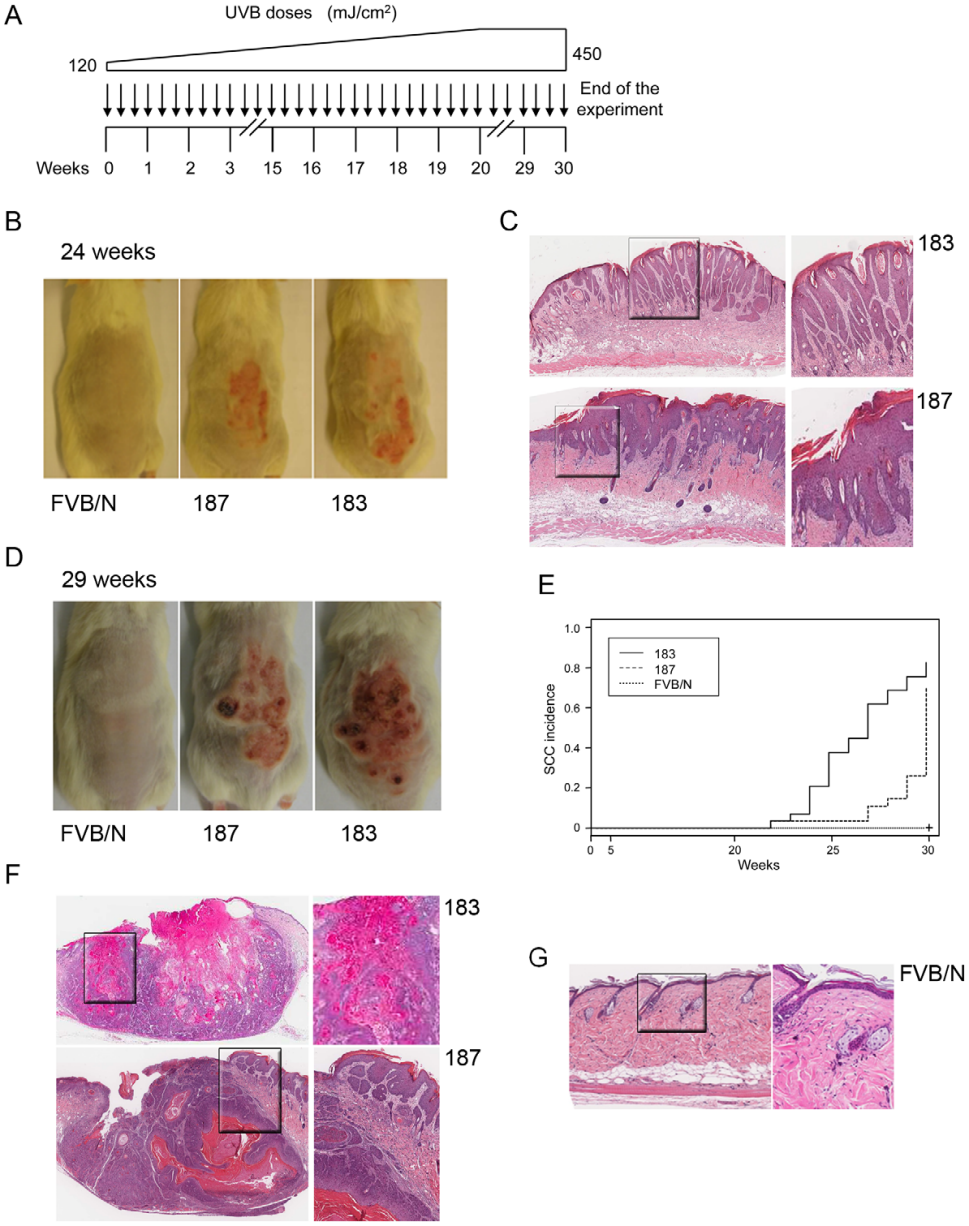
Tumour burden in wild-type and K14 HPV38 E6/E7-Tg animals upon UVB irradiation. (A) Schematic diagram of the experimental procedure of long-term UVB irradiation. (B) Representative pictures of dorsal skin from wild-type FVB/N and HPV-Tg mice exposed to UVB light for 24 weeks. (C) Representative pictures of HE-stained actinic keratosis affected epidermis (AK) from K14 HPV38 E6/E7-Tg mouse lines 183 and 187 after 24 weeks of irradiation (original magnification 10×). Magnified areas are shown in the right panels. (D) Representative pictures of dorsal skin from wild-type FVB/N and HPV-Tg mice exposed to UVB light for 29 weeks. (E) Percentage of animals with skin SCC in wild-type and Tg cohorts of lines 183 and 187. Tumour formation was monitored each week until the end of the experiment in week 30 after start of treatment, and confirmed by histological analyses after sacrifice of the animals. The difference between the curves of control and transgenic mice is statistically significant (*p*<0,0001 determined by logrank test for group data). (F) Representative pictures of HE-stained of SCC sections (SCC) from K14 HPV38 E6/E7-Tg mouse lines 183 and 187 after 30 weeks of UVB irradiation (original magnification 5×). Magnified areas are shown in the right panels. (G) Representative pictures of HE-stained epidermis from wild-type FVB/N mice after 30 weeks of UVB irradiation (original magnification 10×). Magnified area is shown in the right panel.

To evaluate this hypothesis, long-term UVB experiments, in which FVB/N and Tg mice of lines 183 and 187 were exposed to multiple and increasing doses of UVB, were carried out (Figure 6A). After 20–25 weeks of treatment, the majority of the Tg mice from both lines showed thick, scaly, crusty and reddish patches in dorsal skin exposed to the UVB light, while no lesions were observed in wild-type animals (Figure 6B). Histological analyses revealed that these lesions resemble the precancerous condition of actinic keratosis. The representative sections in Figure 6 C show downward prolongations and slight atypia of the rete pegs without stromal invasion (top panels) and parakeratosis, acanthosis and broadened elongated rete ridges with atypia (bottom panels), which are all features of the SCC precursor, actinic keratosis. In later weeks (25–30), SCC become visible on the UV-irradiated skin of Tg mice from both lines, while still no lesions were detected in the control mice (Figure 6D). Histological analysis of skin sections revealed that more than 80% of the Tg animals from line 183 developed SCC during the 30 weeks of UV irradiation (Figure 6E). SSC were also detected in the dorsal skin of line 187 Tg animals, but after a longer latency period than in line 183 (Figure 6E). The different incidence of SCC in the two Tg lines tightly correlates with the HPV38 E6 and E7 expression levels (Figure 1B). Representative images of SSC lesions observed in K14 HPV38 E6/E7 Tg mice after 30 weeks of treatment are shown in Figures 6F. These lesions show the presence of tumour cells with atypia, horn formation (Figure 6F, top panels) and tumour cell invasion deep into the dermis (Figure 6F, bottom panels). In contrast, histological examination of dorsal skin sections from FVB/N animals at weeks 30 evidenced only irritation and slight atypia in the epidermis (Figure 6G).

Together, these data show that HPV38 oncoproteins and UVB irradiation cooperate in the development of actinic keratosis and SCC.

## Discussion

The role of beta HPV types in NMSC is still not conclusively fully established. Functional studies revealed that beta HPV E6 and E7 proteins, similarly to their homologues from the mucosal HR HPV types, have the ability to deregulate fundamental cellular events, such as cell cycle, apoptosis and senescence [20] [21] [22] [23] [24] [32]. However, despite the functional similarities between E6 and E7 from beta and mucosal HR HPV types, the two subgroups of HPV types may induce cancer development by two distinct mechanisms. It is well demonstrated that the mucosal HR HPV types play a key role in cancer initiation and maintenance. In fact, their genomes and E6 and E7 expression are detected in all cervical cancer cells, and inhibition of the expression of these viral oncogenes in those cells resulted in a rapid induction of apoptosis and/or senescence [33]. In contrast, analyses of skin lesions suggest that beta HPV types may be involved only at early stage of skin carcinogenesis. This hypothesis is mainly based on two findings: (i) higher viral load was found in the SCC-precursor lesion AK than in SCC and (ii) not all cancer cells resulted positive for beta HPV DNA [34]. Taking into consideration also the properties of beta HPV oncoproteins to interfere with the regulation of cell cycle and apoptosis, it is conceivable to hypothesize that beta HPV E6 and E7 enhance the carcinogenicity of sunlight, facilitating the accumulation of DNA damages induced by UV and consequently cancer development. In normal cells, DNA damage induced by UV irradiation activates cellular defense processes leading to p53 activation, which in turn induces cell-cycle arrest or apoptosis to allow repair or elimination of the damaged cells, respectively. In contrast in beta HPV infected cells, E6 and E7 expression circumvent the activation of the cellular defense processes by the UV irradiation, maintaining the cells in a proliferative state and allowing efficient viral DNA replication. As a side-effect, these events favour the accumulation of UV-induced DNA damages and cellular transformation. Due to the irreversible nature of the UV-induced damages, e.g. mutation of tumour suppressor genes, the maintenance of the transformed phenotype may become independent of the viral gene expression.

Our data obtained with K14 HPV38 E6/E7 Tg mice support this model. Indeed, while in normal mice UV irradiation led to accumulation of the cell-cycle inhibitor p21^WAF1^ and cell cycle arrest, in the Tg animals these UV-induced phenomena were strongly inhibited. In addition, although HPV38 E6 and E7 expression *per se* did not lead to significant morphological alterations of the epidermis, it strongly facilitated the induction of SCC by chemical carcinogens or chronic UV irradiation. Most importantly, SCC development upon chronic UV exposure of K14 HPV38 E6/E7 Tg mice irradiation was preceded by lesions that closely resemble actinic keratosis, the precursor lesions of SCC also observed in humans.

The different susceptibility of the K10 and K14 HPV38 E6/E7 Tg models to UV-mediated carcinogenesis indicates that the expression of HPV38 E6 and E7 in the basal layer of the epidermis is an essential event for the development of skin cancer induced by chronic UV irradiation. This conclusion is supported by the fact that the natural HPV infection and expression of the viral oncoproteins initiate in the cells of the basal layer. However, it is also possible that the different behavior of the K10 and K14 Tg mice after exposure to chronic UV irradiation is due to the different efficiency of the two keratinocyte promoters, K10 and K14, in expressing the viral genes. Indeed, K10 Tg animals express approximately 3–4 lower levels of HPV38 E6 and E7 than K14 Tg animals (Viariso et al, unpublished data). Thus, additional studies are required to elucidate the different cancer susceptibility of Tg mice expressing the viral oncogenes in the basal or supra-basal layers of the skin. To evaluate the importance of the HPV38 E6 and E7 expression levels in UV-induced carcinogenesis, we are currently generating a novel K14 Tg line that expresses similar levels to the K10 Tg animals.

Previous transgenic models used to study the role of beta HPV in cutaneous cancer express the entire early region (ER) of HPV8 or E6 gene under control of the K14 promoter [25] [26]. The HPV8 ER and HPV8 E6, showed a remarkable similarity in the development of skin lesions. Indeed, a single dose of UV led to a rapid development of papillomas and SCC in both Tg models [26], which also spontaneously developed benign tumours and, in a small percentage, SCC [25] [26]. Although these data support the role of HPV8 in skin carcinogenesis, the HPV8 animal models do not closely correspond to the situation observed in humans, where beta HPV infection is normally asymptomatic and SCC development appears to be strongly associated with chronic UV exposure. The difference observed in K14 HPV8 E6 or HPV8 ER and K14 HPV38 E6/E7 Tg mice may simply reflect the more aggressive properties of the oncoproteins from HPV8 in comparison to HPV38. However, studies in *in vitro* experimental models do not support this hypothesis. In fact, HPV8 E6 and E7 displayed lower *in vitro* transforming activities when compared to the oncoproteins from the mucosal HR HPV types [35] [36] [37]. In contrast, HPV38 E6 and E7 were able to immortalize primary human keratinocytes [22] [23], to deregulate p53 functions [24] and up-regulate the expression of the catalytic subunit of the telomerase hTERT [23] [32], all features shared with E6 and E7 from the mucosal HR HPV types. The different phenotype of the K14 HPV8 ER and K14 HPV38 E6/E7 Tg mice could be explained by a different number of integrated copies of HPV DNA and expression levels of the viral oncoprotein in the two Tg models. In addition, regarding the K14 HPV8 ER Tg mice, it is likely that the product of other early genes cooperate with E6 and E7 in promoting cancer development. For instance, HPV8 E2 has been shown to display transforming properties in *in vitro* and *in vivo* models [38] [39]. However, as already described above, K14 HPV8 E6 and HPV8 ER Tg mice showed a very similar phenotype, indicating that E2 may play a less important role than E6 in the induction of skin lesions [25] [26]. Thus, it is not yet clear why these two animal models, K14 HPV8 E6/E7 and K14 HPV38 E6/E7, showed different phenotypes.

Independently of these differences, both Tg animal models provided evidence for a cooperation of the beta HPV types and UV irradiation in skin carcinogenesis. Interestingly, the cooperation of infectious agents and environmental factors in carcinogenesis has also been shown in previous studies on bovine papillomavirus type 4 (BPV4). In cows grazed on grass or fed on hay, BPV4 infection leads to development of papillomas of the upper gastrointestinal tract that are spontaneously rejected in a relative short time, e.g. 12 months. In contrast, in cows kept on a diet of bracken fern, BPV4-induced papillomas persist and progress to cancer. This phenomenon is explained by the fact that bracken fern contains several molecules that induce immunosuppression or mutagenesis. These immunosuppressants favour the persistence of the viral infection, while mutagenic substances, e.g. quercetin, promote DNA damage, rendering the infected cell more susceptible to transformation. Thus, studies on beta HPV types and BPV4 underline the importance of environmental factors in virus-mediated carcinogenesis.

## Methods

### Plasmid construction and generation of Tg mice

The E6 and E7 ORFs of HPV38 were amplified by polymerase chain reaction (PCR) using as template the entire HPV38 genome, and were cloned in a pGEM-3Z vector containing the K14 promoter, a β-globin intron 2, and the K14 polyadenylation sequence (kindly provided by Professor Herbert Pfister, University of Cologne). The complete insert was isolated (see Figure 1A) and microinjected, at a concentration of 3 ng/µl, into the pronuclei of fertilized eggs to generate Tg mice, as described previously [40]. HPV38 E6 and E7 positivity was determined by PCR using specific primers located in the 5′ (5′-ATG GAA CTA CCA AAA CCT CA-3′) and 3′(5′-TTA TCG TCC GCC ATT GCG-3′) regions of the E6 and E7 genes, respectively.

We identified two lines (183 and 187) of HPV38 E6/E7 Tg mice in a FVB/N genetic background. Experiments were performed with K14 HPV38 E6/E7 transgenic lines 183^tg/wt^, and 187^tg/wt^ and wild-type FVB/N littermates. The animals were kept in the central animal unit of the DKFZ, Heidelberg, Germany, under an artificial day/night rhythm and were fed Kliba 3437 standard food pellets and water *ad libitum* if not stated otherwise.

### Ethics statement

All experiments described in this study were performed in strict accordance to federal law and the standard ethical guidelines (NIH, 1985; European Communities Directives, 1986 86/609/EEC) and approved by local government authorities (Regierungspräsidium Karlsruhe, Germany) under license G162-08. Animal treatments, e.g. UV irradiation, were performed under Sevorane anesthesia, and all efforts were made to minimize suffering.

### Total RNA isolation and reverse transcription PCR analyses

Total RNA was isolated from dorsal skin, ear, esophagus, tongue, and liver of 6–8-week-old Tg animals using the Qiagen RNeasy isolation kit (Quiagen, Hilden, Germany). cDNAs were synthesized from 1 µg of total RNA using the M-MLV reverse transcriptase (Invitrogen, Darmstadt, Germany), 18 bp length polydT were used as primers. Quantitative reverse transcription PCRs (RT-qPCRs) were performed in a 25 µl mixture containing 1 µl of 1∶5 diluted cDNA and SYBR-green master mix (SA bioscience, Frederick, Maryland) with specific HPV38 E6 primers (5′-TGC TTA TGC TTC TGC TCA ATA TG-3′ and 5′-GTC TGT TGC TCC ACC TGT TC-3′) or mouse GAPDH primers to amplify a housekeeping gene as internal control (5′ –AAG AAG GTG GTG AAG CAG GCA TC-3′ and 5′-CGA AGG TGG AAG AGT GGG AGT TG-3′), using an Applied Biosystems 7300 machine (Applied Biosystems, Darmstadt, Germany). The fluorescence threshold value was calculated using the SDS analysis software from Applied Biosystems.

### Histological and immunohistochemical analysis

Tissue samples from 6–8-week-old mice were fixed in 4% formaldehyde for 24 h at room temperature, and embedded in paraffin. Five µm sections were either stained with hematoxylin/eosin (HE) or used for immunostaining using anti Ki-67 (1∶200) (MM1, Novocastra, Wetzlar, Germany) or p21^WAFI^ antibody (1∶250) (556431, BD Pharmingen, Heidelberg, Germany). Staining was performed using biotin-labeled goat anti-mouse immunoglobulin G and ABC agent from M.O.M kit (Vector Peterborough, UK). The percentages of positive cells were determined by counting 400 hematoxylin-stained cells under 40× magnification in four different fields of the epithelium.

For histological analyses, tissue samples from wild-type and Tg mice were fixed in 4% formaldehyde for 24 h at room temperature, and embedded in paraffin and five µm sections were stained with hematoxylin/eosin (HE).

### UVB treatments

UVB irradiation was performed with a Bio-Spectra system (Vilber Lourmat, Marne La Vallee, France) at a wavelength of 312 nm. Each animal was anesthetized with 3% Sevorane (Abbott, Wiesbaden, Germany) in an inhalation anesthetizer (Provet, Lyssach, Switzerland) and placed in a covered compartment with an upper square opening (3×2 cm) at a distance of 40 cm from the UVB lamp. To determine the impact of viral proteins on UV-induced checkpoints, 7-week-old mice where shaved on the dorsal skin, and irradiated up to 5 times in a row, maximum 2 times a day, with UVB at 450 mJ/cm^2^. Twenty-four hours after the last dose, mice were sacrificed and skin sections stained as described above.

To study UV-induced carcinogenesis, groups of n = 30 7-week-old female FVB/N wild-type or transgenic mice of lines 183^tg/wt^ and 187^tg/wt^ were shaved on the dorsal skin with electric clippers and irradiated 3 times a week for 20 weeks with increasing doses of UVB, starting from 120 mJ/cm^2^ to a final dose of 450 mJ/cm^2^, with a constant weekly increase to allow skin thickening. For the following 10 weeks mice were irradiated 3 times a week with 450 mJ/cm^2^. The tumour incidence (tumour bearers/group) was recorded weekly. Tumours were identified first macroscopically and by histological diagnosis. After thirty weeks, or earlier if the tumour reached the ethically allowed maximal size, the animals were sacrificed and HE-stained sections of dorsal skin served for histological diagnosis.

### Initiation-promotion experiments

Five weeks after birth, mice were shifted to Altromin 1324 diet. For epicutaneous applications of initiator, the dorsal skin was shaved with electric clippers 7 days before treatment. Experimental groups of n = 20 (acetone/acetone) or n = 23–27 (7,12-dimethylbenz-anthracene (DMBA)/12-O-tetradecanoyl-phorbol-13-acetate (TPA)) 7-week-old female FVB/N wild-type or transgenic mice of lines 183^tg/wt^ and 187^tg/wt^ were initiated either by a single epicutaneous application of 0,2 ml acetone or 400 nmol DMBA in 0.2 ml acetone. Beginning one week later, the mice were treated each twice weekly with 0.1 ml acetone or with 5 nmol TPA in 0.1 ml acetone for maximally 20 weeks. Papilloma and carcinoma development was monitored up to week 24 without further treatment. Animals were monitored every three days throughout the experiment. The tumour incidence (tumour bearers/survivors in percent) and yield (number of tumours/survivors) were recorded weekly. Tumours were first identified macroscopically and later on by histological diagnosis.

### Statistical analysis

Percentages of positive cells in immunostained sections were compared between the different lines with the Student's t-test and statistical analysis were performed with GraphPad Prism (version 4.00, GraphPad Software Inc., La Jolla, CA, USA) Time to first tumor, time to death and time to SCC were displayed with Kaplan-Meier plots or 1- Kaplan-Meier plots. Animals sacrificed for analysis were considered censored. Time to first tumor, time to death and time to SCC were compared between the different groups with the logrank test. For the CoCa experiment, maximal tumor burden was determined for every animal and groups were compared with the Wilcoxon rank sum test. Statistical analyses were performed with R (version 2.12.0, Copyright (C) 2010 The R Foundation for Statistical Computing) and SAS (Version 9.2, SAS Institute Inc., Cary, NC, USA).
