# Correction: E6 and E7 from Beta Hpv38 Cooperate with Ultraviolet Light in the Development of Actinic Keratosis-Like Lesions and Squamous Cell Carcinoma in Mice

**DOI:** 10.1371/journal.ppat.1006005

**Published:** 2016-10-28

**Authors:** Daniele Viarisio, Karin Mueller-Decker, Ulrich Kloz, Birgit Aengeneyndt, Annette Kopp-Schneider, Hermann-Josef Gröne, Tarik Gheit, Christa Flechtenmacher, Lutz Gissmann, Massimo Tommasino

Fig 1 is incorrect. The original figure does not show the presence of two loxP elements immediately before and after the viral genes. The authors have provided a corrected Fig 1 here.

**Fig 1:**
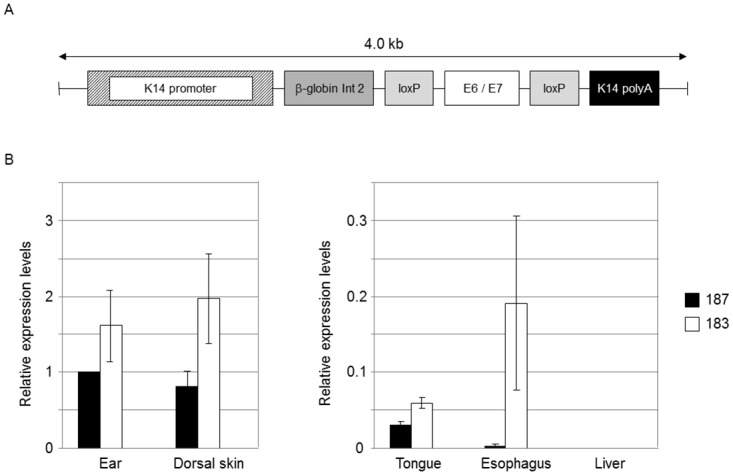
HPV38 E6 and E7 expression in Tg mice. (A) Schematic representation of the K14-HPV38 E6/E7 construct. (B) HPV38 E6 and E7 transcripts are differentially expressed in the epithelia of the two hemizygous Tg mouse lines 183, and 187. Total RNA was extracted from the ear, the skin, tongue, esophagus, and liver. After preparation of cDNA, E6 and E7 expression was determined by RT-qPCR and normalized towards the expression level of GAPDH. The data shown in the Figures are the means ±SD of three independent experiments. In each experiment the 187 ear data is set to 1 and the other values are consequently resized.
